# Integrated analysis from multi-center studies identities m7G-derived modification pattern and risk stratification system in skin cutaneous melanoma

**DOI:** 10.3389/fimmu.2022.1034516

**Published:** 2022-12-01

**Authors:** Xin Zhang, Ying Miao, Hao-Wen Sun, Yi-Xiao Wang, Wen-Min Zhao, A-Ying Pang, Xiao-Yan Wu, Cong-Cong Shen, Xiao-Dong Chen

**Affiliations:** ^1^ Department of Dermatology, Affiliated Hospital of Nantong University, Medical School of Nantong University, Nantong, China; ^2^ Department of Gastroenterology, Affiliated Hospital of Nantong University, Medical School of Nantong University, Nantong, China

**Keywords:** N7-methylguanosine, skin cutaneous melanoma, immune microenvironment, prognosis, immunotherapy

## Abstract

The m7G modification has been proven to play an important role in RNA post-transcriptional modification and protein translation. However, the potential role of m7G modification patterns in assessing the prognosis of Skin cutaneous melanoma (SKCM) and tumor microenvironment (TME) has not been well studied. In this study, we investigated and finally identified 21 available m7G-related genes. We used hierarchical clustering (K-means) to classify 743 SKCM patients into three m7G-modified subtypes named m7G/gene cluster-A, B, C. We found that both m7G cluster B and gene cluster B exhibited higher prognosis and higher immune cell infiltration in TME compared to other subtypes. EIF4E3 and IFIT5, two m7G related genes, were both markedly elevated in Cluster B. Then, we constructed an m7G score system utilizing principal component analysis (PCA) in order to evaluate the patients' prognosis. High m7G score subtype was associated with better survival prognosis and active immune response. Overall, this article revealed that m7G modification patterns were involved in the development of the tumor microenvironment. Evaluating patients' m7G modification patterns will enhance our understanding of TME characteristics and help to guide personal treatment in clinics in the future.

## Introduction

Skin cutaneous melanoma (SKCM) is a malignant tumor originating from melanocytes and is one of the most lethal human tumors. Around 25% of melanomas are transformed from the original nevus (1) and sunlight exposure, especially strong intermittent exposure patterns, is also an important environmental factor that increases the risk (2). Patients with early-stage melanoma can be cured by surgery. But patients with advanced melanoma are still unsatisfactory, with a 5-year survival rate of about 27% (3). Because metastatic melanoma is not sensitive to conventional chemoradiotherapy (4), the treatment of melanoma remains a great challenge.

In recent years, emerging immunotherapy has become an important means of treating melanoma, such as anti-CTLA4 antibodies and anti-PD1/L1 antibodies, which were called immune checkpoint inhibitors (ICIs). Among them, anti-PD1/L1 antibodies are more widely used and can effectively improve the prognosis by increasing infiltrating CD8+ T cells (2, 5). ICIs have significantly improved progression-free survival and overall survival in some patients, however, for most patients, ICIs are associated with low overall response rates. Although the identification of PD-L1 expression can screen patients with an immune response to PD-L1 antibodies, PD-L1 expression is not recommended as a predictor of immune response, as individual heterogeneity results in the inconsistency between PD-L1 expression levels and clinical benefits (5). Recent studies have shown that specific components of the TME, especially the activation of CD8+ T cells, upregulate the expression of immunosuppressive factors such as PD-L1 through a negative feedback regulatory mechanism of immunity (6). This means that immunotherapy may preferentially benefit patients with substantial CD8+ T infiltration in the TME. Therefore, predicting response to ICIs based on the characteristics of TME is a critical step to improve response to existing ICIs therapy (7).

RNA methylation is a common form of epigenetic modification, including m6A, m1A, m5C, m7G, etc., according to the different methylation sites (8). m7G refers to the addition of a methyl group to guanosine at the N7 position of the RNA ribosome. m7G exists not only in the 5’ cap region of mRNA but also in mRNA, tRNA, and rRNA, which plays an important role in the maintenance of normal physiological functions of the human body (9). m7G is involved in almost every stage of mRNA life cycle including transcription, splicing of pre mRNA, nuclear export, and translation (10–13). In recent years, more and more studies have shown that m7G-related genes play an important role in the pathogenesis of tumors. As the most studied methyltransferase involved in m7G-related processes, METTL1 usually forms a complex with WDR4, and its overexpression is often associated with some malignant tumors such as intrahepatic cholangiocarcinoma (14). This is mainly because the METTL1/WDR4 complex can increase the m7G modification of a subset of tRNAs, thereby reducing ribosomal pausing and increasing the translation efficiency of cancer-promoting mRNAs such as EGFR, which drives cancer development (14–16). Furthermore, the expression of METTL1 correlates with tumor drug resistance. Okamoto et al. (17) showed that knockdown of NSUN2 and METTL1 genes enhanced the sensitivity of HeLa cells to 5-FU, which provides a new perspective to address the mechanisms of resistance to cancer chemotherapy drugs. Ago2 is also involved in the m7G methylation process of RNA, forming a complex by assembling with microRNA, inhibiting the initiation of mRNA translation by binding to the m7G cap of targeted mRNA, precluding the recruitment of eIF4E and inhibiting the migration of lung cancer cells (18, 19).

At present, there are few studies on m7G modification, and the relationship between m7G and TME is still unclear. Most of the studies are limited to 1-2 m7G regulators, therefore, a comprehensive analysis of multiple m7G regulators will deepen our understanding of the TME.

Therefore, in this paper, a comprehensive analysis of m7G-related genes was performed through the malignant melanoma transcriptomic and genomics sequencing database. Three m7G modification patterns and gene subtypes were established by unsupervised clustering and the relationship between each subtype and the prognosis of SKCM patients and immune cell infiltration in the tumor microenvironment were analyzed. In addition, we also constructed an m7G scoring model using PCA to quantify the m7G modification pattern of individuals and used it to explore the potential relationship between this scoring model and survival prognosis, immune response, and TME. The establishment of a Nomogram helps to guide better prediction of patient’s survival prognosis in clinical. In conclusion, our finding suggests that m7G modification plays a crucial role in the tumor immune microenvironment formation and in predicting patient prognosis and immunotherapy efficacy.

## Materials and methods

### Data acquisition and preprocessing

The RNA-Seq (Level-3 HTseq-FPKM) sequencing data of all SKCM patients were downloaded from the TCGA database (20) and 3 repeated sequencing samples from the same patient were excluded. Finally 465 non-repeated tumor samples were included. At the same time, the RNA-Seq data of 557 normal skin samples from the GTEx project were downloaded as normal controls. After excluding non-coding RNAs, they were standardized with tumor samples for difference analysis. In addition, the GSE53118, GSE65904, and GSE78220 datasets were downloaded from the GEO database (21), and gene annotation was performed on the respective platform files as a validation cohort. In the survival analysis, no survival status was excluded and samples with an overall survival time of less than 1 day were modeled and validated. Finally, 454 SKCM patients were included in TCGA-SKCM, 79 SKCM patients were included in GSE53118 and 210 SKCM patients were included in GSE65904. It is worth noting that GSE78220 is an anti-PD-1 immunotherapy cohort, including a total of 27 SKCM patients, in which clinical information includes the corresponding situation of immunotherapy. In addition, the copy number variation (CNV) and somatic mutation data of SKCM were downloaded from the TCGA database. It is worth mentioning that in the TCGA-SKCM cohort, RNA-seq data in FPKM format were converted to TPM. The “ComBat” algorithm in the “sva” package was used to eliminate batch effects in the TCGA and GEO databases (22) and three cohorts indicated above were integrated to establish a Meta cohort. m7G related genes were obtained from the existing literature (23) and related gene sets GOMF_m7G_5_PPPN_DIPHOSPHATASE_ACTIVITY,GOMF_RNA_CAP_BINDING, GOMF_RNA_7_ METHYLGUANOSINE_CAP_BINDING. m7G-related genes: DCP2, IFIT5, EIF3D, EIF4G3, NSUN2, GEMIN5, AGO2, NUDT10, EIF4E, EIF4E2, NCBP2, NUDT11, NUDT3, NCBP1, METTL1, LARP1, NUDT4, EIF4E3, SNUPN, WDR4, LSM1, NUDT16, DCPS, CYFIP1.

### Unsupervised clustering

An unsupervised consensus clustering analysis was performed based on m7G regulators or m7G pattern-regulated gene expression levels. Principal component analysis (PCA) to determine whether each subtype is relatively independent of the other subtypes. The number of clusters was determined by the R package “conensusClusterPlus” (24), and 100 replicates were performed with pltem=0.8 to verify the stability of the subtypes. Kaplan Meier curves were used to evaluate overall survival (OS) of different SKCM patients in the dataset and log-rank test was used. We performed PCA analysis to reduce the dimensionality, judging the ability of distinguishing patients.

### Calculation of m7G score

First, we normalized the differentially expressed genes (DEGs) extracted from different m7G clusters and extracted overlapping DEGs, we used Cox regression method to perform prognostic analysis on each overlapping DEG and screened genes at P<0.05. Principal component analysis (PCA) was used to construct the m7G cluster signatures. Both principal components 1 and 2 are selected as feature scores, m7Gscore=∑(PC1i+PC2i).

### Enrichment analysis

Differences in biological pathways between subtypes were assessed using gene set variation analysis (GSVA) (25). Gene Ontology (GO) is used to annotate the biological processes, molecular functions, and cellular components of genes (26). Gene pathways were annotated using the Kyoto Encyclopedia of Genes and Genomes (KEGG) (27). Differential genes between different subtypes were analyzed using the “limma” package (p < 0.05) (28), and then the overlapping genes among the three groups were analyzed by GO and KEGG using the “clusterProfiler” package. In addition, c2.cp.kegg.v7.0.symbols.gmt was used as the reference gene set, and FDR < 0.05 was the screening threshold.

### Drug sensitivity analysis

IC50s were calculated using the prophetic package in R software, and chemotherapeutic drugs were obtained from the genome of Drug Sensitivity in Cancer (GDSC) database.

### Immunoassay

In immune cell analysis, we simultaneously used different algorithms, such as TIMER, CIBERSORT, QUANTISEQ, MCP-counter, XCELL, and EPIC, to estimate the abundance of immune cells in different samples (29). In addition, the ESTIMATE algorithm was used to calculate the immune score, and the interstitial score to reflect the microenvironmental status.

### Statistical analysis

Correlation coefficients between immune cells and m7G regulator expression were calculated by Spearman correlation analysis. The Kruskal-Wallis test was used for differences among the three groups, and the χ2 test was used for associations between categorical covariates. Based on the correlation of m7Gscore with patient prognosis, the optimal cutoff value for each dataset subset was defined using the “survminer” R package. This value divided patients into high and low m7Gscore subgroups. The log-rank statistic is used to reduce batch effects of calculations. OS maps were drawn using the Kaplan-Meier method and the log-rank test was used to identify statistical differences. Univariate Cox regression was used to calculate hazard ratios for m7G regulators and genes associated with m7G phenotypes. Multivariate Cox regression was used to identify independent survival factors. The “Maftools” package and its “oncoplot” function were used to present mutational differences (30). P<0.05 was considered statistically significant.

## Results

### Genetic variation profile of m7G-related genes in SKCM

**Figure 1:**
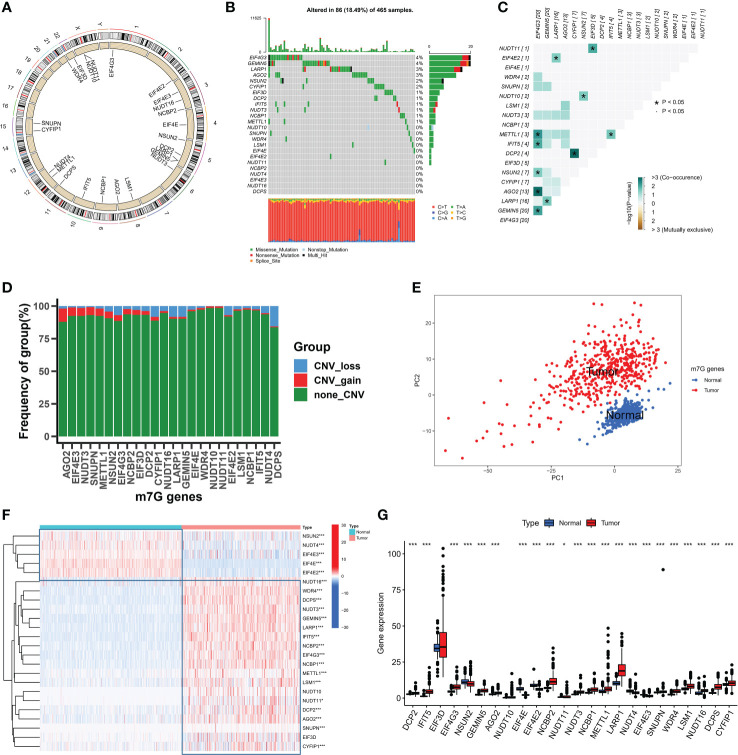
The landspace of genetic and variation of 24 m7G genes in SKCM. **(A)** The location of 24 m7G genes on chromosomes. **(B)** The mutation frequency of 24 m7G regulators in 465 patients, with each color representing the mutant types. Numbers on the right part of the figure represent the mutation frequency. **(C)** Co-occurrence and exclusion of 24 m7G genes mutations. Green color represents co-occurrence, and yellow color represents exclusion. **(D)** The CNV frequency of 24 m7G genes. The red color represents amplification as well as the blue color represents deletion. **(E)** Principal component analysis of m7G genes. **(F, G)** The expression levels of 24 m7G genes in normal and tumor tissues. (*p < 0.05;***p < 0.001).

In this study, a total of 24 m7G-related genes were identified in the TCGA cohort and the locations of m7G-related genes on chromosomes are shown in **Figure 1A**. Firstly we summarized the frequency of copy number variation (CNV) and somatic mutation of m7G-related genes in SKCM. EIF4G3 and GEMIN5 had the highest mutation frequency out of 465 samples, with 86 mutated with a frequency of 18.49 percent (**Figure 1B**). Missense mutations were common. Additionally, strong mutational co-occurrence links between EIF4G3, METTL1, IFIT5, MSUN2, AGO2, and GEMIN5 were discovered in the co-mutation map of 24 m7G regulators (**Figure 1C**). According to the frequency of CNV modifications, AGO2 focused on copy number amplification, CNV deletion frequencies in DCPS were common, and CNV alterations were common in m7G-related genes (**Figure 1D**). Combining SKCM samples in the TCGA database with normal skin samples in the GTEx database, it was discovered that based on the expression of m7G-related genes, SKCM samples could be completely distinguished from normal samples using PCA (**Figure 1E**). To determine whether the aforementioned genetic variants affect the expression of m7G-related genes in SKCM patients, we investigated the mRNA expression levels of m7G-related genes between normal and SKCM samples. With the exception of EIF3D, most of the m7G-related genes showed significantly different expression levels between samples. Compared with SKCM samples, NSUN2, NUDT4, EIF4E3, EIF4E and EIF4E2 showed higher expression in normal tissues (**Figure 1F, G**). The above analysis showed that m7G-related genes were highly heterogeneous between normal and SKCM samples, suggesting that the imbalanced expression of m7G-related genes plays a crucial role in the occurrence and progression of SKCM.

### Modification patterns mediated by m7G-related genes

**Figure 2:**
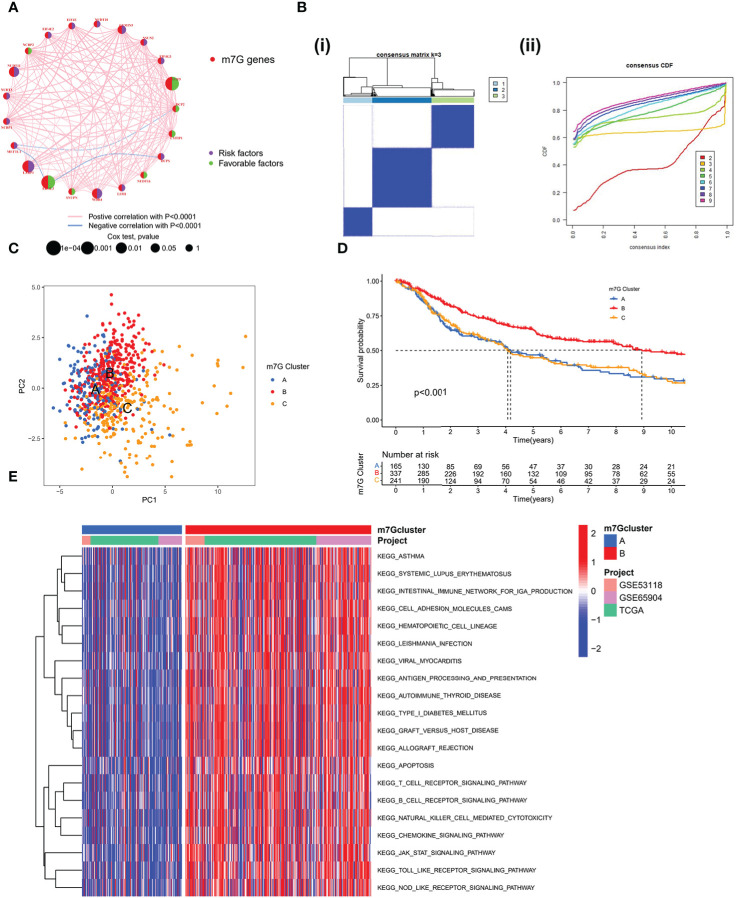
Patterns of m7G methylation modification. **(A)** Interaction network of the 21 m7G genes. In meta-SKCM cohort(GSE53118, GSE65904, TCGA). Purple dots represent risk factors and green dots represent favorable factors. **(B)** 743 SKCM patients were divided into 3 groups using Consensus clustering matrix. **(C)** Principal component analysis of m7G modification pattern. **(D)** Kaplan–Meier survival analysis of OS for different m7G clusters in meta-SKCM cohort(p < 0.001). **(E)** GSVA enrichment analysis between m7G cluster A and B.

A Meta cohort was created by combining three datasets (GSE53118, GSE65904, and TCGA-SKCM) with complete prognostic data. 21 m7G-related genes were subsequently annotated in the Meta cohort. The Kaplan-Meier survival analysis and log-rank test were used to separate the prognostic significance of 15 m7G-related genes for SKCM patients using the optimal cut-off value for each group (**Figure S1A**). Low expression of other genes was linked to better prognostic outcomes, with the exception of high expression of EIF4E3, IFIT5, and CYFIP1, which exhibited a greater survival advantage. The m7G-related gene network outlines a thorough picture of gene interactions and their prognostic consequences for individuals who have the gene (**Figure 2A**). Most m7G-related genes, according to our research, demonstrated a substantial association and had good predictive capacity (univariate cox regression analysis). And IFIT5 and EIF4E3 were significantly positively correlated with patient survival. According to the results mentioned above, distinct m7G methylation modification patterns may be significantly influenced by crosstalk between m7G-related genes. We used the “ConsensusClusterPlus” R package to classify the patients based on the expression of m7G-related genes; when the K value is 3, the slope of CDF decline was the smallest (**Figure 2B**). And finally three modification patterns were identified, which we refer to m7G cluster-A, B, and C respectively. PCA showed that three patterns were relatively discrete (**Figure 2C**), indicating that SKCM patients could be divided into three clusters based on the expression of m7G-related genes. Prognostic analysis revealed a considerable advantage in the m7G cluster B (**Figure 2D**), but no apparent survival difference between m7G cluster A and C. To explore the biological behavior between different m7G ​​modification patterns, we performed a GSVA enrichment analysis on the meta-SKCM cohort. As shown in **Figure 2E**, compared with m7G cluster A, cluster B presented enriched pathways associated with immune activation, such as T cell receptor signaling pathway, B cell receptor signaling pathway, chemokine signaling pathway and cytokine-cytokine receptor interaction. In addition, KEGG pathways also varied among different modifications. The purine and pyrimidine metabolic pathways were substantially more active in m7G cluster C than in cluster B. When compared to cluster C, cluster A had a considerably higher concentration of the TGF-βmetabolic pathway (**Figure S1B, C**). The results indicated above may provide some evidence support for the prognostic advantage of m7G cluster-B.

### Infiltration characteristics of TME cells under different modification patterns

**Figure 3:**
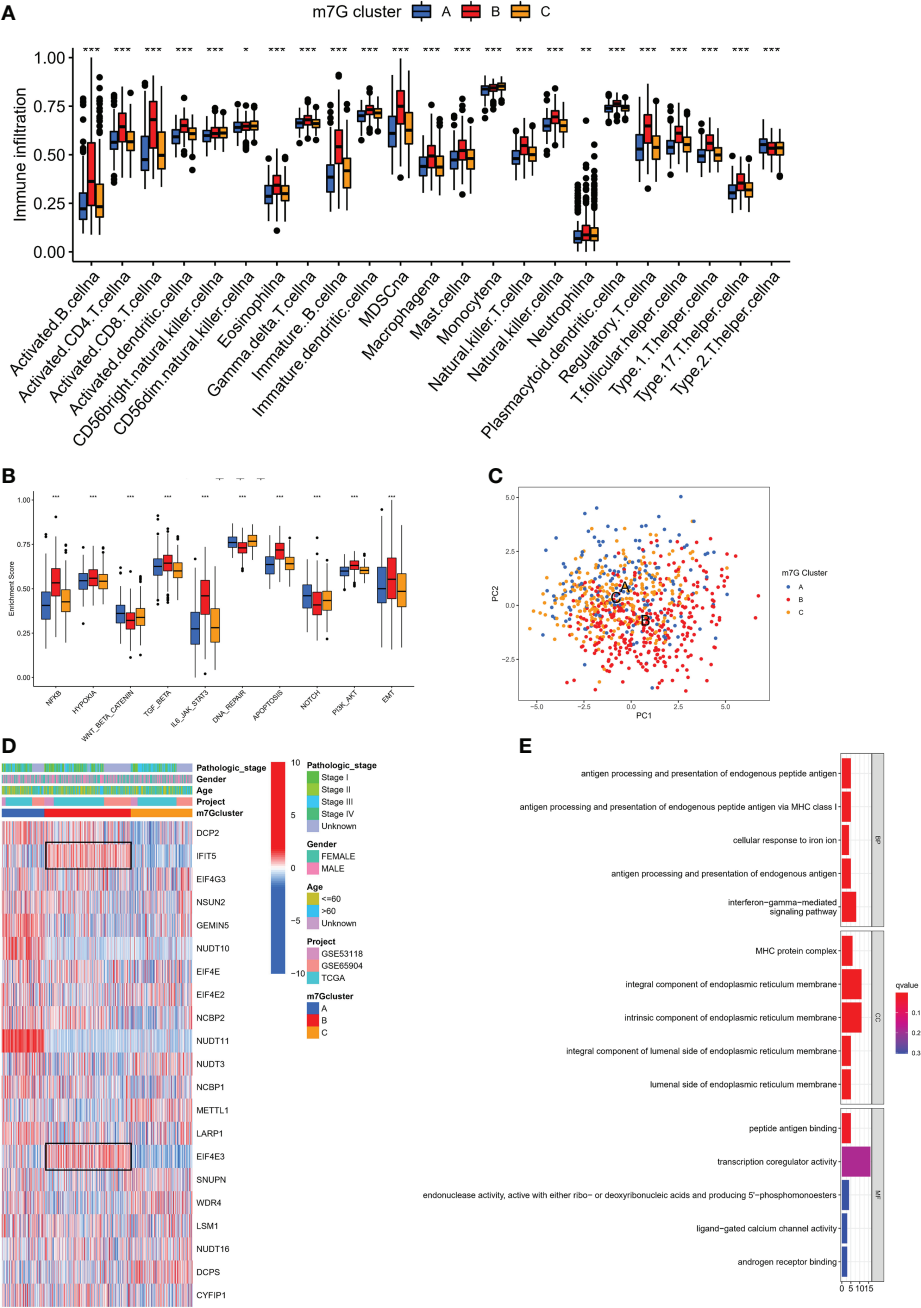
The characteristics of TME in distinct m7G clusters. **(A)** The infiltration level of immune cells in three m7G clusters. (*p < 0.05; **p < 0.01; ***p < 0.001). **(B)** GSVA enrichment analysis based on Hallmark gene set according to different m7G clusters. (***p < 0.001). **(C)** Principal component analysis based on pathway activity score corresponding to m7G clusters. **(D)** Unsupervised clustering of gene expression of 21 m7G genes in the meta-SKCM cohort.M7G cluster, pathologic stage, gender, and age were used as annotations. **(E)** GO enrichment analysis of prognosis-related DEGs.

We used single sample GSEA (ssGSEA) to analyze immune cell infiltration on various m7G clusters in light of mounting evidence that TME plays a significant role in tumorigenesis and progression (31). To our surprise, analysis of the TME showed that m7G ​​cluster B is very abundant in immune cell infiltration, including CD4+ T cells, CD8+ T cells, NK cells, macrophages, eosinophils, mast cells, Myeloid-derived suppressor cells (MDSCs) and dendritic cells, while m7G clusters A and C were dominated by type 2 helper T cells (Th2) and monocytes respectively (**Figure 3A**). GSVA on Hallmarker gene set (32) revealed different biological behavior of three m7G modification clusters. NF-KB pathway and IL6-JAK-STAT3 pathway activity were significantly enhanced in m7G cluster B. In contrast, m7G cluster A was predominantly and significantly enriched in the Wnt-β-catenin signaling pathway and m7G cluster C was characterized by DNA repair-related pathways (**Figure 3B**). PCA analysis also showed that three m7G modification patterns could also be distinguished based on cancer activity pathway scores (**Figure 3C**). In addition, the heatmap showed that among three subtypes, IFIT5 and EIF4E3 genes were significantly up-regulated in m7G cluster-B, NUDT10 and NUDT11 were significantly up-regulated in m7G cluster A and METTL1 was mainly up-regulated in m7G cluster-C (**Figure 3D**).

### The m7G-related DEGs in SKCM

**Figure 4:**
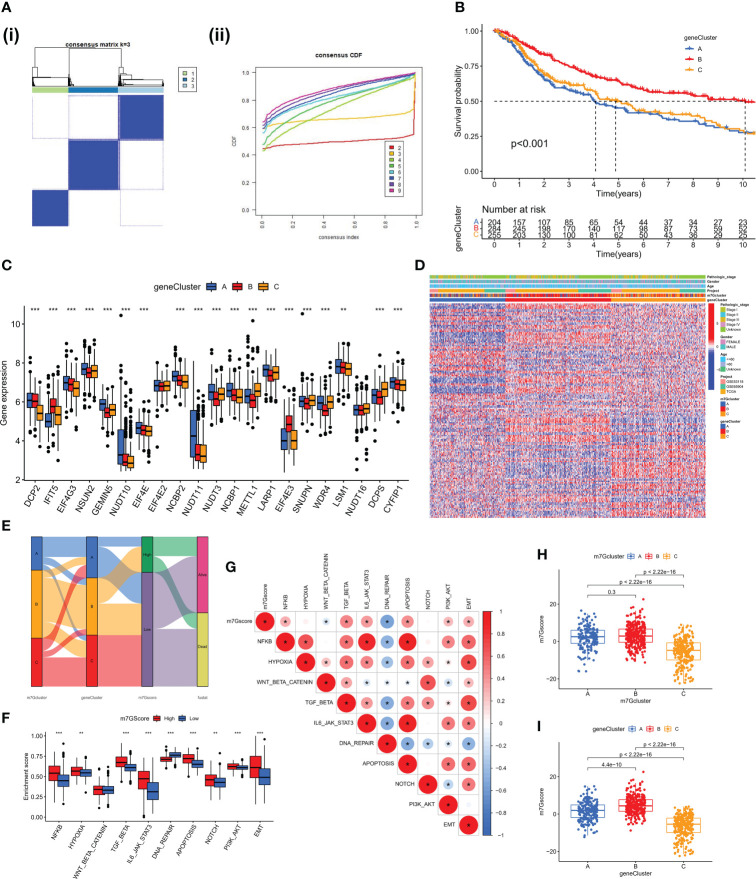
Construction of gene clusters and m7G score based on the DEGs. **(A)** DEGs were divided into 3 groups using Consensus clustering matrix. **(B)** K-M survival analysis of OS for different m7G gene clusters in meta-SKCM cohort(p < 0.001). **(C)** Histogram showing expression of m7G related genes in different gene clusters. The lines in the boxes mean median value. **(D)** Unsupervised clustering of gene expression of 21 m7G genes in the meta-SKCM cohort. Gene cluster, pathologic stage, gender, and age were used as annotations. **(E)** Alluvial diagram showing an association between m7G cluster,m7G gene cluster, m7G score, and survival status. **(F)** GSVA enrichment analysis based on Hallmark gene set according to high and low m7G scores. (**p < 0.01; ***p < 0.001). **(G)** Spearman analysis displaying the correlations between m7G score and hallmark gene set. Red represents positive correlation, blue represents negative correlation. **(H, I)** The difference of m7G score in distinct m7G clusters (above) and gene clusters (below). *represents p < 0.05.

We discovered 248 differentially expressed genes (DEGs) associated with the m7G phenotype using the “limma” package and carried out GO enrichment analysis on DEGs using the “clusterProfiler” tool to further explore the potential biological function of the m7G modification pattern. Surprisingly, these genes showed enrichment for biological processes significantly associated with immune infiltration, confirming that m7G ​​modification plays a non-negligible role in the immune regulation of the TME (**Figure 3E**). To further validate this regulatory mechanism, we performed an unsupervised clustering analysis based on the prognostic-relevant DEGs in order to classify patients into different genotypes. Consistent with the grouping of m7G modification patterns, an unsupervised clustering algorithm revealed three distinct gene subtypes, named gene cluster-A, B, and C (**Figure 4A**). We observed that gene cluster B had the best survival prognosis and gene cluster A, C had no significant difference in survival prognosis (**Figure 4B**). In addition, among three gene subtypes, significant differences in the expression of m7G regulators were observed, which is consistent with the expected results of the m7G methylation modification pattern, while the m7G-related genes EIF4E3, IFIT5 were significantly upregulated in gene cluster B (**Figure 4C, D**).

### The m7G score for individual SKCM patients

The aforementioned analysis is based only on the population and cannot accurately predict the pattern of m7G methylation modification in each patient. Considering the individual heterogeneity of m7G modification, we constructed the m7G score system using PCA algorithm to systematically quantify the m7G modification pattern of SKCM patients based on these phenotype-related genes. All patients with SKCM were classified into high and low groups based on the m7G cut-off value. The alluvial diagram shows the connections between the subtypes (**Figure 4E**). The ssGSEA algorithm analysis showed that the activity of hallmark pathway was significantly enhanced in high score patients (**Figure 4F**). Meanwhile, the analysis of related pathway activity showed that high score may be strongly associated with the heightened activation of the NF-KB and IL6-JAK-STAT3 pathways (**Figure 4G**). The Kruskal-Wallis test revealed that there was a significant difference in m7G score between m7G clusters, with the highest score in m7G cluster B (**Figure 4H**). There was also a significant difference in m7G score between gene clusters, with the highest score in gene cluster B (**Figure 4I**). However, immunological activation was more pronounced in m7G cluster B and gene cluster B. Therefore, the results above strongly suggest that high m7G score is significantly associated with immune activation and m7G Score system can better assess m7G modification patterns in individual patient.

### Prognostic value of m7G score in individual patient

**Figure 5:**
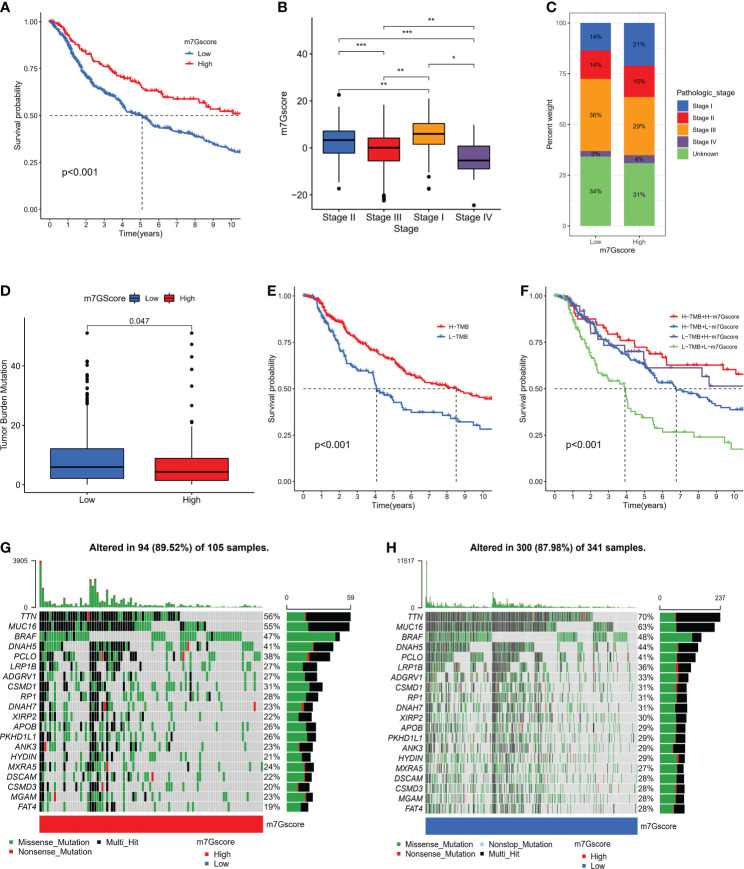
The connection between m7G score and clinical characteristics. **(A)** K-M survival analysis of OS in high and low m7G score groups in meta-SKCM cohort (p < 0.001). **(B, C)** The association between m7G score and clinicopathologic stage. (*p < 0.05 **p < 0.01 ***p < 0.001). **(D)**The difference of TMB in high versus low m7G score(p=0.047). **(E)** K-M survival analysis of OS in high and low TMB subgroups in the meta-SKCM cohort (p <0.001). **(F)** M7G score combined with TMB better predicted the prognosis of SKCM patients. **(G, H)** Mutational landscape of significantly mutated genes (SMGs) stratified by high (left panel) versus low m7G score (right panel) subgroups. Mutation types were used in different colors as annotations.

We further specified the value of m7G Score system in predicting patient outcomes. Patients with high m7G Score demonstrated a substantial survival benefit (**Figure 5A**), whereas patients with advanced disease demonstrated a lower m7G Score (**Figure 5B, C**). In addition, we discovered that the m7G Score had greater survival discriminating value in several clinical subgroups, including various age groups (**Figure S2A**) and sex groups (**Figure S2B**). The m7G Score system also has a good prognostic value in patients with different pathological stages, especially stage II and III (**Figure S2C**). Given that TMB is clinically significant in directing immunotherapy in SKCM patients, we sought to explore the intrinsic correlation between TMB and m7G Score. It was found that the TMB score was slightly higher in the low m7G Score group (**Figure 5D**) and the Low-TMB group represented a worse prognostic outcome (**Figure 5E**). Dividing SKCM patients into four subgroups based on m7G score and TMB, we found that low m7G score combined with Low-TMB indicated worse prognostic outcomes (**Figure 5F**). Then, we used the “maftools” package to analyze the differences in the distribution of somatic mutations between low m7G Score and high m7G Score in the TCGA-SKCM cohort (33), in which TTN was the most widely mutated gene in both groups, while the high m7G Score group had a TTN mutation rate of 56% (**Figure 5G**), while the TTN mutation rate in the low m7G Score group was 70% (**Figure 5H**).

### The role of m7G score in anti-PD-1/L1 immunotherapy

**Figure 6:**
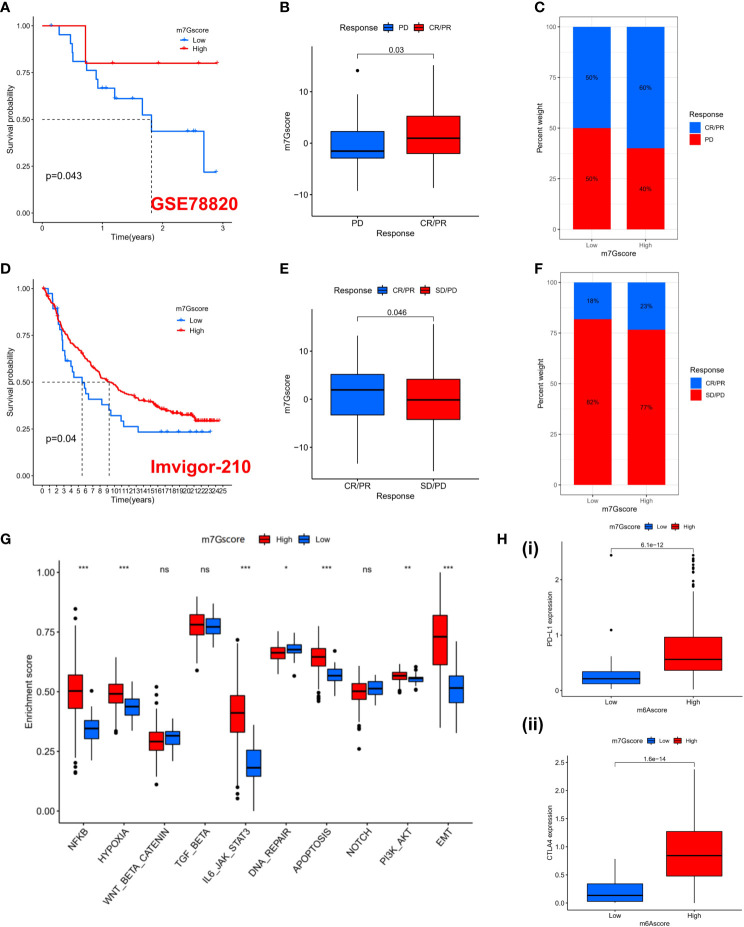
The prediction to Immunotherapy using m7G score. **(A)** K-M survival analysis of OS in high and low m7G score groups in the GSE78220 cohort (p = 0.043). **(B)** Distribution of m7G scores between immune response and non-response in the GSE78220 cohort. (p=0.03). **(C)** The percentage of patients with different responses to immune therapy in the GSE78220 cohort. **(D)** K-M survival analysis of OS in high and low m7G score groups in the IMvigor210 cohort (p = 0.04). **(E)** Distribution of m7G scores between immune response and non-response in the IMvigor210 cohort. (p=0.046). **(F)** The percentage of patients with different responses to immune therapy in the IMvigor210 cohort. **(G)** GSVA enrichment analysis based on Hallmark gene set according to high and low m7G scores in the PD-L1 cohort. (*p < 0.05 **p < 0.01 ***p < 0.001). **(H)** PD-L1 **(i)** and CTLA4 **(ii)** expression levels between high and low m7G score subgroups. NS, no significance; CR, complete response; PR, partial response; SD, stable disease; PD, progressive disease.

Previous results suggest that m7G ​​modification patterns can influence immune cell infiltration. Therefore, we hypothesized that the immune response to anti-PD-1/PD-L1 may be significantly mediated by the differential modification pattern of m7G. Immunotherapy represented by PD-L1 and PD-1 blockade has undoubtedly become a major breakthrough in cancer treatment. We examined the potential predictive value of m7G modification signatures for immunological response to immune checkpoint blockade based on two immunotherapy cohorts (GSE78220 and IMvigor210). GSE78220 is the anti-PD-1 immunotherapy cohort, while IMvigor210 is the anti-PD-L1 immunotherapy cohort. In the anti-PD-1 cohort, patients with high m7G score showed significant clinical benefit and significantly prolonged OS (**Figure 6A**). Immunotherapy outcomes in patients with high m7G score were more inclined to CR/PR (**Figure 6B, C**). Likewise, in the anti-PD-L1 cohort, patients with high m7G Score had better prognostic outcomes (**Figure 6D**) and were more prone to CR/PR (**Figure 6E, F**). The aforementioned content suggests that quantification of m7G modification patterns is a potential and reliable biomarker for predicting prognosis and assessing therapeutic effectiveness of immunotherapy. Subsequently, we predicted the hallmark pathway activity level in the anti-PD-L1 cohort and found that, similar to the m7G Cluster, patients with high m7G scores had more notable immune activation (**Figure 6G**). In addition, the expression levels of PD-L1 and CTLA4 were also significantly up-regulated in patients with high m7G score (**Figure 6H**). In conclusion, our research strongly demonstrates that m7G ​​methylation modification patterns are highly associated with tumor immunophenotype and response to anti-PD-1/L1 immunotherapy. And the established m7G modification signature will aid in the prediction of the anti-PD-1/L1 response to immunotherapy.

### Indicative role of m7G score in the immune microenvironment

**Figure 7:**
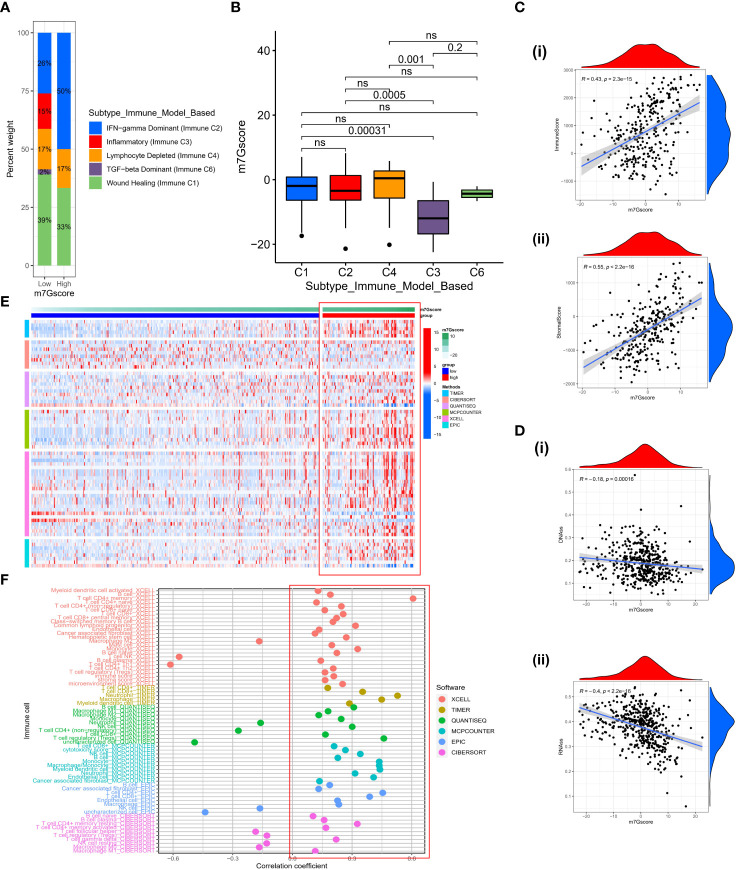
The characteristics of TME in high and low m7G score subgroups. **(A)** The fraction of Immune Subtypes in high and low m7G score subgroups. **(B)** The distribution of m7G score between different Immune subtypes. **(C)** The association between immune score **(i)** or stromal score **(ii)** and m7G score. **(D)** The relationship between DNAss **(i)** or RNAss **(ii)** and m7G score. **(E)** Heatmap of immune cell infiltration level based on TIMER, CIBERSORT, QUANTISEQ, MCP-counter, XCELL, EPIC algorithms. **(F)** The correlation between m7G score and activity of immune cells in TME. ns, no significance.

Based on the gene expression profiles of all solid tumors in the TCGA, Thorsson et al. identified five immune-expression signature subtypes: Wound Healing (Immune C1), IFN-gamma Dominant (Immune C2), Inflammatory (Immune C3), Lymphocyte Depleted (Immune C4) and TGF-beta Dominant (Immune C6) (34). Based on the aforementioned results, we discovered that immunological subtypes varied considerably between different m7G score groups and that “IFN-gamma Dominant” predominated (**Figure 7A**), representing a higher proportion of lymphocytes Infiltration in the high m7G score group. Significant differences in the m7G score between various immunological subtypes were also observed (**Figure 7B**). The ESTIMATE algorithm also demonstrated that as the m7G score is increased, the immune score and stromal score are also increased (**Figure 7C**), indicating that high m7Gscore was associated with high immune cell infiltration and high stromal cell levels. We conducted a correlation analysis of the stemness scores for DNA and RNA in patients with SKCM in light of the crucial role that stemness plays in tumor formation and treatment (35). Unsurprisingly, stemness scores decreased as m7G scores increased. (**Figure 7D**). To comprehensively explore the relationship between different m7G ​​score groups and the immune microenvironment, we calculated the immune cell infiltration level of each patient in the TCGA-SKCM cohort based on six algorithms (TIMER, CIBERSORT, QUANTISEQ, MCP-counter, XCELL, and EPIC). It was discovered that the TME was in an active state and there was higher immune cell infiltration in the group with high m7G scores (**Figure 7E**). Similarly, the majority of immune cells were positively correlated with m7G score (**Figure 7F**). Although most literature reported that SKCM is not susceptible to radiotherapy and chemotherapy, we explored whether m7G score could have a certain indicative effect on traditional cytotoxic drugs. Therefore, we used the “prophetic” package to calculate the IC50 values ​​of various drugs. With the exception of Docetaxel, it was found that high m7G score groups were more sensitive to chemotherapy drugs (**Figure S3**).

### Construction of nomogram based on m7G score

We developed a nomogram based on m7G score since the nomogram can be applied intuitively to clinical practice to assess the prognosis of patients. To construct the nomogram, we incorporated the statistically relevant indicators from the multivariate Cox regression, such as the m7G score, Age, and Stage (**Figure S4A**). For example, in the low m7Gscore population, patients with a total score of 58.4 in stage I and older than 60 years old had a probability of survival of 0.042 for less than one year, 0.225 for less than three years, and 0.333 for less than five years (**Figure S4B**). According to ROC curve, nomogram had good predictive performance for survival in TCGA and GEO cohorts (GSE53118, GSE65904) (**Figure S4C, D**). In addition, calibration curve results for 1-, 3-, and 5-year survival showed that actual survival was close to predicted survival in the TCGA-SKCM cohort (**Figure S4E**). In the GEO cohort (GSE53118, GSE65904), the result is the same as the aforementioned content (**Figure S4F**).

## Discussion

Due to the presence of variable loops, tRNAs are the most common m7G-modified RNA species (36). The m7G modification of tRNA is important for normal mRNA translation and maintenance of embryonic stem cell self-renewal. If damaged, it can lead to the progression of microcephaly primitive dwarfism and cancers (37). Previous studies have shown that m7G modification selectively promotes the regulation of cell cycle and translation of oncogenic mRNAs, which correlates with the number of codons decoded by m7G-modified tRNAs (16). In addition to intrahepatic cholangiocarcinoma, abnormal m7G modification is also associated with esophageal squamous cell carcinoma, acute myeloid leukemia, glioblastoma multiforme, and breast cancer (14, 36). However, the relationship between m7G modification and melanoma is unclear. Therefore, this article aims to explore the relationship between multiple m7G-related genes and TME in order to better predict patient prognosis and guide more effective immunotherapy strategies.

First, we identified three distinct modification patterns based on 21 m7G-related genes with distinct biological behaviors and prominent TME infiltration characteristics. Tumors were classified into three immunophenotypes: immunological-inflammatory, immune-desert, and immune-excluded based on the immune background of the tumor (31). The immune-inflammatory type refers to the infiltration of CD4+T, CD8+T and other immune cells in the tumor parenchyma, which is related to the inflammatory response (38). Immuno-excluded means that immune cells are surrounded by a matrix and cannot penetrate into the parenchyma (39). Immune desert type refers to the lack of infiltration of relevant immune cells in both the parenchyma and stroma of the tumor (31, 39). GSVA showed that m7G ​​Cluster B significantly enriched immune activation-related pathways such as T and B cell receptor signaling pathways and m7G Cluster B had higher infiltration of adaptive immune cells and macrophages in TME. The levels of these immune cells directly affect the onset of the adaptive immune response and correlate with a patient survival advantage (6, 40, 41). m7G Cluster A was significantly enriched for matrix-related pathways, such as TGF-β. TGF-β suppresses immune responses by limiting T cell infiltration into tumors (42). m7G Cluster C is associated with immune-oncogenic pathways such as DNA repair. There is a tendency to classify Cluster B as an immune-inflammatory phenotype and m7G Clusters A and C into an immune-desert phenotype.

Then we further investigated the DEGs associated with the m7G phenotype to further explore the potential biological functions of these genes. GO enrichment analysis showed that DEGs were significantly associated with immune-related biological pathways and were identified as three genomic subtypes, indicating that m7G ​​modification is important in shaping the TME. In addition, JAK/STAT3 signaling was upregulated in cluster B, and previous study reported that JAK/STAT3 signaling enhances PD-L1 expression (43), therefore they can response to PD-L1 therapy better than cluster A and C. What’s more, we found that EIF4E3 and IFIT5 were significantly up-regulated in Cluster B regardless of m7G modification grouping or gene grouping and were associated with high survival prognosis of patients. We reasonably suspect that EIF4E3 and IFIT5 may enhance antitumor activity by promoting the activation of immune responses. IFN-induced tetratricopeptide repeat protein 5 (IFIT5), a member of the IFIT family, is an important enhancer of the innate immune response, initiating several immune signaling pathways to defend itself, including IRF3, NF-kB (44, 45). IFIT5 has a special tetratricopeptide repeat (TPR) structure that regulates cell function by recognizing its partner to form a complex, affecting cell migration ability and proliferative activity (46, 47). Studies have reported that high expression of IFIT5 is associated with more immune cell infiltration and its low expression is an independent risk factor for the prognosis of patients with malignant melanoma (48), which is consistent with the conclusion of this paper. Furthermore, IFIT5 plays different roles in the development of certain tumors. For instance, by preventing the transformation of microRNA (miRNA), IFIT5 can increase the expression of EMT transcription factors and increase the risk of developing renal cell carcinoma (49). EIF4E3 is a member of the eukaryotic translation initiation factor EIF4E family, which affects mRNA processing, nuclear export, translation and cancer development by specifically recognizing the 5′m7G cap structure of mRNA. Unlike other EIF4E family members in their cancer-promoting roles (50, 51), EIF4E3 competes with EIF4E1 for the same transcriptional and translational targets, such as VEGF, cyclinD1, through atypical binding to the cap and hinders tumor development by reducing the expression of these factors, which is important. tumor suppressor and was confirmed in AML (52, 53).

Afterwards, based on DEGs, we established an m7G scoring system to better assess the heterogeneity of individual m7G modification patterns. Although high TMB showed better survival and was often associated with better immune responses in patients receiving immunotherapy, our results showed lower TMB in the high m7G score subgroup, suggesting that the m7G scoring system can be a more effective predictor of patient prognosis than TMB. We also noticed that the somatic mutation rate of TTN was highest in the low subgroup, with a statistically significant difference between the two groups. Studies have shown that TTN deficiency can down-regulate cell cycle-related proteins such as Cyclin D1, CDK2 and up-regulate apoptosis-related proteins, which may be related to the regulation of its upstream long non-coding RNA TTN-AS1 (LncRNA-TTN-AS1). Hypomethylation of the transcription initiation site leads to overexpression of lncRNA-TTN-AS1, which increases TTN expression by activating promoters upstream of TTN and promotes tumor proliferation and migration (54). This may provide a new therapeutic target and therapeutic strategy for malignant melanoma.

In recent years, only a minority of the population has benefited from immunotherapy, so we sought to assess whether the m7G score could serve as a novel biomarker to predict patient responses to immunotherapy. Two independent immunotherapy cohorts confirmed the predictive power of the m7G score in anti-PD-1/L1 immune response, with a significantly higher response rate in the high m7G score subgroup than in the low group. We also noticed that NF-KB signal transduction pathway in the high m7G score subgroup was significantly active in the PD-L1 cohort. Studies have demonstrated that in patients who have seen a full or partial response to immunotherapy, the codon G34E mutation in the NF-KB inhibitor epsilon (NF-KBIE) causes loss of NF-KBIE function and activation of the NF-KB signal transduction pathway. Overactivated NF-KB pathway allows patients to better benefit from immunotherapy and will promote the maturation of dendritic cells and recruit more CD8+ T cells (55, 56). In addition, we also explored the potential relationship between m7G score and TME and found a higher degree of immune cell infiltration of TME in high m7G score subgroup. This suggests that different m7G ​​modification patterns can have a huge impact on TME shaping. Subsequently, we established a nomogram, combined with a variety of clinical indicators, to establish a personalized prognostic prediction scale to quantify multiple risk factors of different individuals. These results suggest that the m7G score can be used to guide immunotherapy regimens and assess patient prognosis.

However, there are some limitations in this paper. Firstly, although this paper integrates 21 m7G-related genes, only using retrospective data may introduce some bias. Therefore, our next goal is to collect more samples from patients with melanoma and verify the molecular mechanism of m7G-related gene regulation through relevant experiments. In addition, due to the small number of immunotherapy cohorts, more data on patients receiving ICI treatment in medical centers will be further collected in the future to establish a prospective study.

## Conclusion

In conclusion, we comprehensively assessed the characteristics of the tumor microenvironment with different m7G modification patterns and the results indicated that m7G modification plays an important role in regulating immune responses. Importantly, in three m7G modification patterns, Cluster B was associated with high survival prognosis of patients, which showed significant upregulation of m7G-related genes, EIF4E3 and IFIT5. We also established an m7G scoring system, which can be an effective predictor of patient prognosis. These m7G-related genes may play a role as a prognostic biomarker for patient resistant or sensitive to immunotherapy. Assessing the m7G modification pattern of patients will better guide the immunotherapy regimen and improve the overall survival rate of patients in the future.

## Data availability statement

The datasets presented in this study can be found in online repositories. The names of the repository/repositories and accession number(s) can be found in the article/**Supplementary Material**.

## Author contributions

XZ contributed to this work. YM and H-WS conceptualized and designed this study. All authors contributed to the article and approved the submitted version.

## Funding

The work was supported by Key Medical Research Projects of Jiangsu Provincial Health Commission (No. ZD2022006), the Science and Technology Project of Nantong City (No. JC22022083) and Postgraduates of Jiangsu Province (SJCX21_1455).

## Conflict of interest

The authors declare that the research was conducted in the absence of any commercial or financial relationships that could be construed as a potential conflict of interest.

## Publisher’s note

All claims expressed in this article are solely those of the authors and do not necessarily represent those of their affiliated organizations, or those of the publisher, the editors and the reviewers. Any product that may be evaluated in this article, or claim that may be made by its manufacturer, is not guaranteed or endorsed by the publisher.
