# Supplementary material for: Integrated analysis from multi-center studies identities m7G-derived modification pattern and risk stratification system in skin cutaneous melanoma
Source: Front Immunol. 2022 Dec 1;13:1034516. doi: 10.3389/fimmu.2022.1034516 (PMC9751814; doi:10.3389/fimmu.2022.1034516)
Supplement: Supplementary file 1 [file DataSheet_1.docx]

Supplementary Material

# Supplementary Figures and Tables

## Supplementary Figures


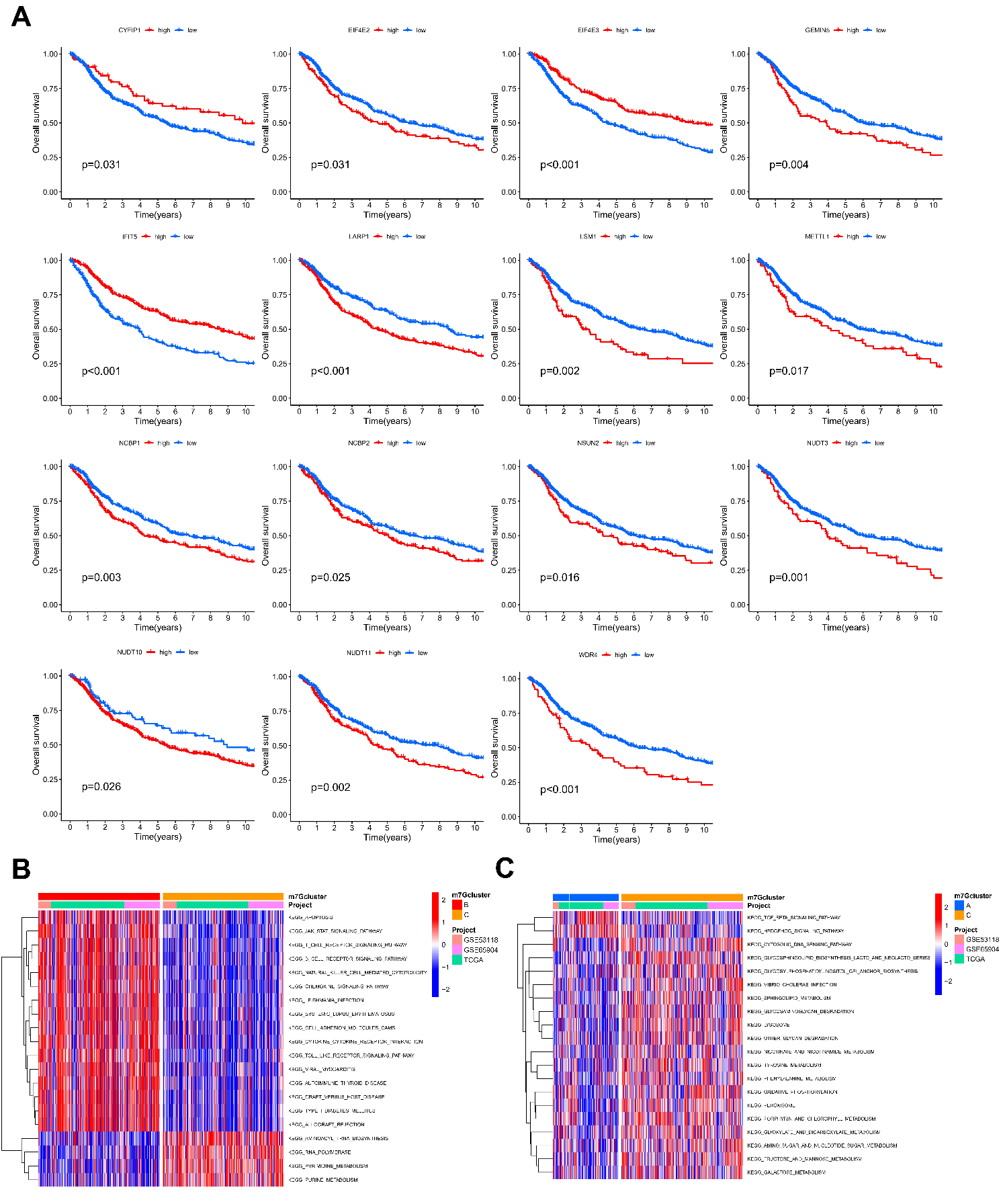


**Supplementary Figure S1.** GSVA enrichment analysis in three m7G clusters. (A) K-M survival analysis of OS stratified by 15 m7G related genes. (B-C) GSVA enrichment analysis describes the distinct biological functions between m7G clusters. (B) m7G cluster B VS C. (C) m7G cluster A VS C.


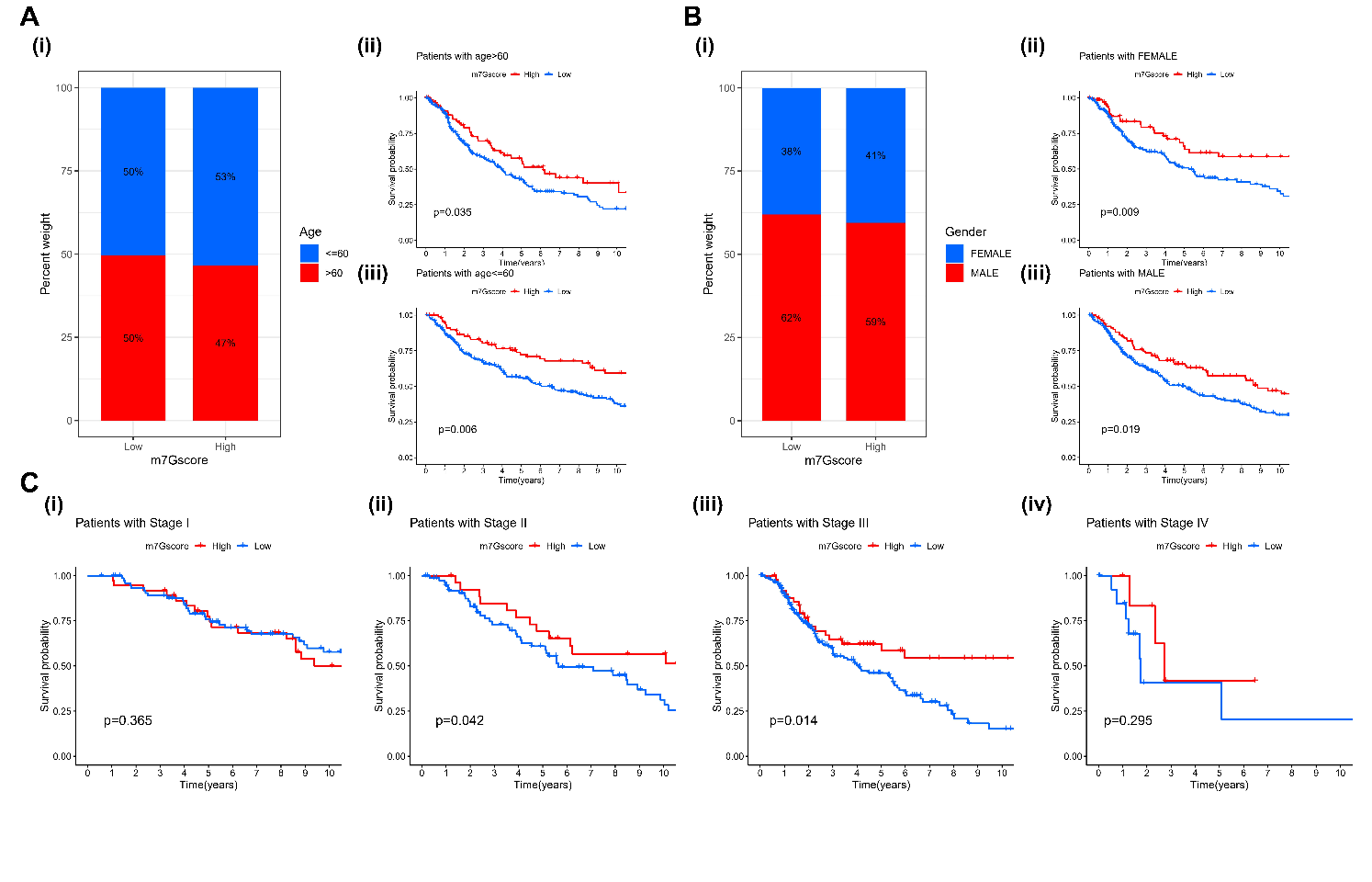


**Supplementary Figure S2**. The risk stratification of m7G score system. (A-C) K-M survival analysis of OS stratified by clinical features in high and low m7G score subgroups. (A) Patients with age >60 and Patients with age ≤60 (B) Female patients and Male patients (C) Patients with stage I，II，III，IV.


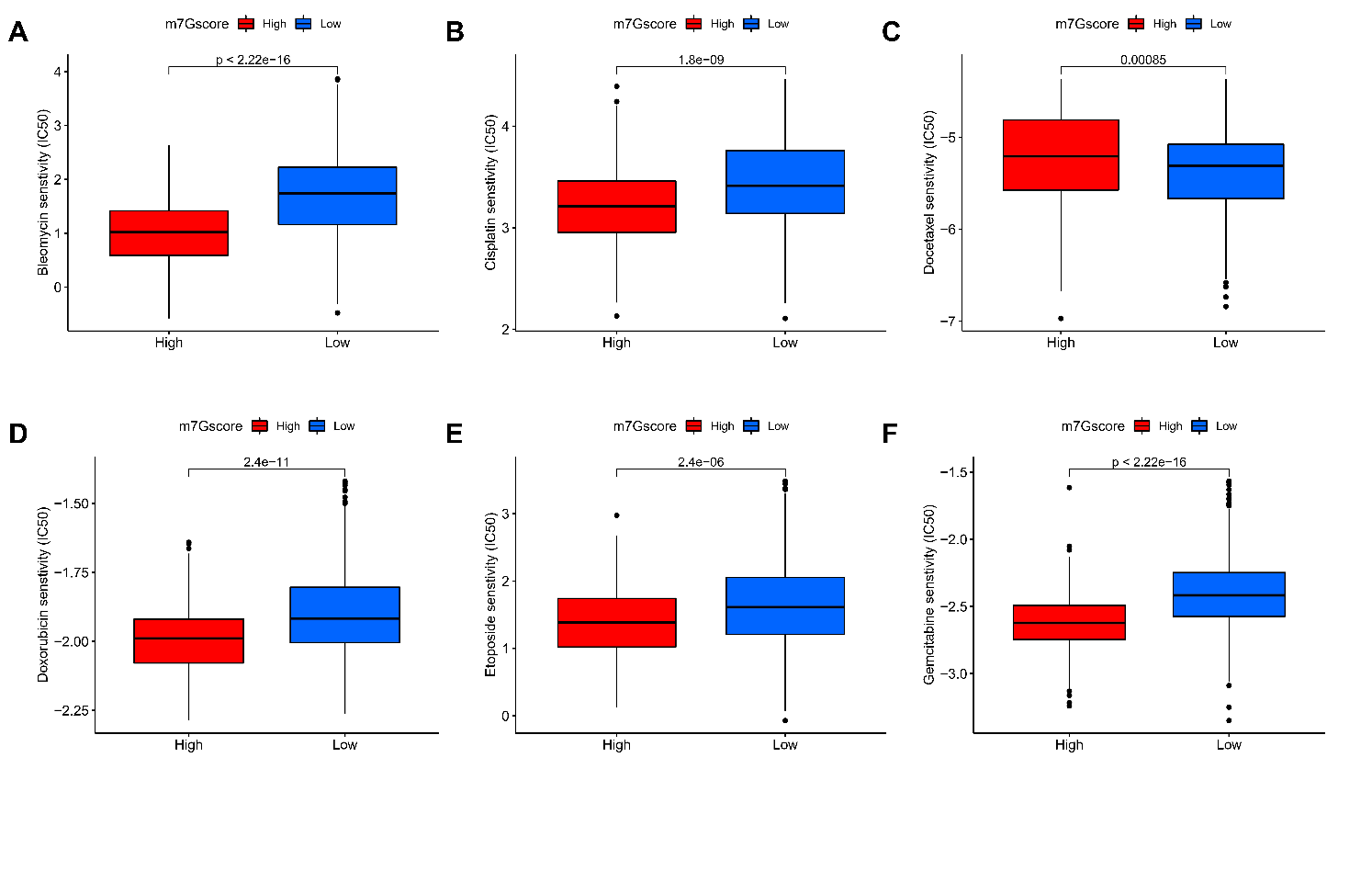


**Supplementary Figure S3.** IC50 of traditional chemotherapeutic Agents in high and low m7G score subgroups. (A) Bleomycin. (B) Cisplatin. (C) Docetaxel. (D) Doxorubicin. (E) Etoposide. (F) Gemcitabine.


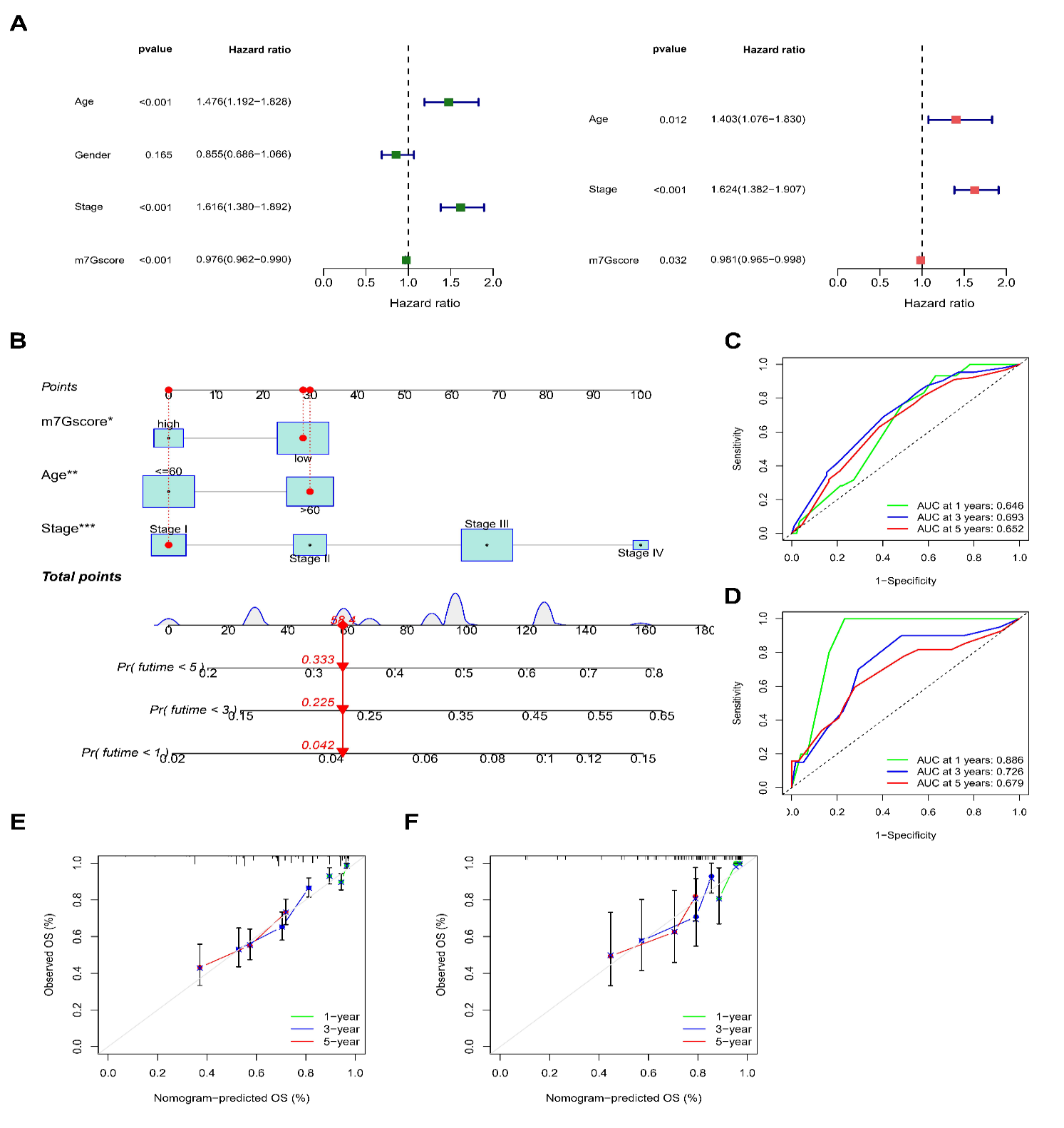


**Supplementary Figure S4.** The construction of nomogram and validation of efficacy prediction in SKCM. (A) Univariate and multivariate Cox regression analysis to screen out independent prognostic risk factors. (B) The nomogram to predict the 1-, 3-, and 5-year survival probability. (C-D) ROC curve indicating the efficacy prediction of m7G score in GSE53118 cohort (C) and GSE65904 cohort (D). (E-F) The calibration curve shows a good predictive value of patients at 1-, 3-, and 5-year in TCGA-SKCM cohort (E) and GEO cohort (F). The abscissa represents the predicted OS and the ordinate represents the actual OS. 95% confidence interval is indicated by the vertical line.
